# Predictors of parents’ infant vaccination decisions: A concept derivation

**DOI:** 10.4102/hsag.v26i0.1697

**Published:** 2021-09-30

**Authors:** Eloïse Botha, Daleen van der Merwe, Rosemary J. Burnett, Petra Bester

**Affiliations:** 1 Africa Unit for Transdisciplinary Health Research, Faculty of Health Sciences, North-West University, Potchefstroom, South Africa; 2 Department of Virology, School of Medicine, Sefako Makgatho Health Sciences University, Pretoria, South Africa

**Keywords:** consumer decisions, consumer health, Health Belief Model (HBM), infant vaccination, primary preventive healthcare, vaccine hesitancy

## Abstract

The myths surrounding coronavirus disease 2019 (COVID-19) vaccines have prompted scientists to refocus their attention on vaccine hesitancy, which is fuelled by the spread of misinformation. The scientific investigation of behavioural concepts relating to vaccine hesitancy can be enhanced by the examination of behavioural concepts from the field of consumer sciences. South African consumer scientists study personal decisions that contribute to individuals’ well-being, including the decisions to prevent ill health. Current data on the predictors of vaccination decisions do not incorporate consumer science constructs imperative in decision-making, which could provide fresh insights in addressing vaccine hesitancy. This study aimed to investigate and illustrate the analogy between concepts of the Health Belief Model (HBM) as parent model, and consumer behaviour that could affect parents’ infant vaccination decisions, by applying a concept derivation approach. The HBM was analysed within the context of public health, including literature from consumers’ vaccination decisions, medical decisions, paediatrics, vaccinology, virology and nursing. Through a qualitative, theory derivation strategy, six main concepts of the HBM were redefined to consumer sciences, using four iterative concept derivation steps. Concept derivation resulted in consumer behaviour concepts that could be possible predictors of parents’ infant vaccination decisions, including consumers’ values; risk perception; consideration of immediate and future consequences; self-efficacy; cues to action; demographics; personal information and knowledge. These predictors could be a starting point for a context- and product-specific consumer primary preventive healthcare decisions model. Our findings highlight the opportunities for interdisciplinary collaboration in investigating consumer primary healthcare-related behaviour.

**Contribution:**

This study introduced interfaces between consumer science and health science literature. Through interdisciplinary collaboration, a better understanding of influences to promote primary preventive healthcare can be achieved.

## Introduction

The coronavirus disease 2019 (COVID-19) pandemic and consequent vaccine developments shed new light on vaccine myths and the long-standing phenomenon of vaccine hesitancy. Vaccine hesitancy was listed in 2019 as one of the top 10 global health threats (World Health Organization 2019), despite the fact that vaccination eradicated smallpox, and drastically reduced the incidence of vaccine-preventable diseases (VPDs), thus saving 2 million – 3 million lives every year (Rodrigues & Plotkin 2020). Consumers base vaccination decisions increasingly more on misinformation spread through the internet and social media (Stecula, Kuru & Hall Jamieson 2020). This misinformation results in vaccine hesitancy, that is, delay or refusal of available vaccination services (MacDonald & Sage Working Group on Vaccine Hesitancy 2015), reduction in vaccination coverage (Dube et al. 2018; Larson 2018) and outbreaks of VPDs in countries that had previously eliminated these diseases.

Health science researchers have addressed the need to investigate individuals’ behaviour in health-related contexts, including the uptake of vaccinations, by employing concepts fundamental to consumer science. Within South Africa, patients are consumers from a legal perspective (considering the Consumer Protection Act 68 of 2008) within a dichotomous health system (Rowe & Moodley 2013), with a nascent National Health Insurance system and increased focus on healthcare quality. In terms of vaccination decisions specifically, authors like Betsch et al. (2018) and Larson (2018) contributed immensely towards understanding these behaviours. However, these behavioural studies which were conducted in non-consumer science disciplines may be enhanced by an examination of consumer behaviour concepts.

Consumer science evolved internationally as a discipline encompassing a diverse range of specialisations enhancing individual and family well-being, including food and nutrition, family studies, apparel, and fashion (McGregor 2011). More recently, consumer science studies have focused on consumer well-being, protecting consumers’ interests through policy and regulation, and consumer information and education (McGregor 2013). Currently, consumer behaviour to promote consumer well-being is being emphasised (Du Preez 2017). Also, consumer scientists have conducted research relating to primary preventive healthcare decisions concerning food choices incorporated into lifestyle (Seme et al. 2017). Therefore, the call for consumer scientists to expand their expertise into the neglected area of health-related consumer research (Van der Merwe 2017) is timely and necessary.

The data on predictors of vaccination decisions in high-income countries are available (Betsch et al. 2018), but based on models that do not incorporate consumer science constructs imperative in decision-making. The identification of context-specific predictors of vaccination decisions based on consumer science may provide fresh insights in addressing the problem of vaccine hesitancy amongst consumers.

We approached this topic from the perspective of the Health Belief Model (HBM) by Hochbaum and Rosenstock (1974) using infant vaccination decisions as a point of departure. Owing to a lack of consumer science literature on vaccination decisions, we explored disciplines from health sciences for predictors of these decisions. The HBM, a model developed in the United States of America in the 1950s and still used in public health today to explain and predict health behaviour, was identified as a parent model to identify predictors to be included in consumer science primary preventive healthcare decision research. The HBM is also frequently applied as a conceptual framework in vaccination-related studies (Guvenc, Seven & Akyuz 2016; Skinner, Tiro, & Champion 2015; Wong et al. 2021). Based on this background, the researchers asked, ‘How can the concepts of the HBM be transposed to consumer behaviour that may affect consumers’ infant vaccination decisions?’ This study aimed to investigate and illustrate the analogy between concepts of the HBM and those from consumer behaviour that could affect consumers’ infant vaccination decisions, by applying concept derivation.

## Theoretical approach: The health belief model

The HBM aimed to explain health behaviours of those failing to undergo screening tests for early detection of disease, or to take preventive measures such as vaccinations against diseases such as tuberculosis, polio and influenza, despite their being free of charge or at a meagre cost (Rosenstock 1974). The underlying assumptions of the HBM for activating health-related behaviours in persons are *firstly*, the perception that an adverse health condition can be avoided; *secondly*, believing that by following specific advice, a harmful health condition can be averted; and *thirdly*, being convinced of one’s capability of adhering to the recommended behaviour. Therefore, the HBM aims to motivate people towards positive health actions to avoid adverse health outcomes and depicts individuals’ beliefs as mediators for their actions.

**FIGURE 1:**
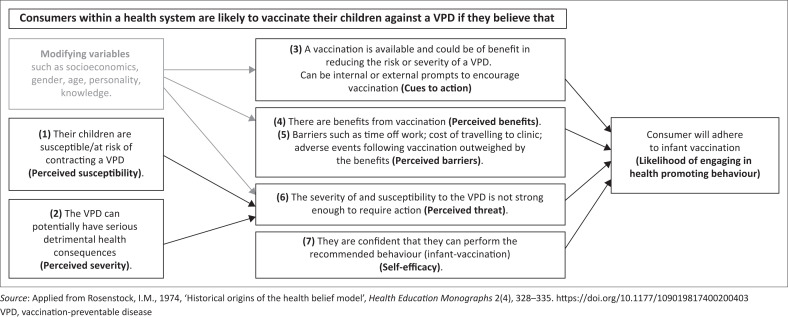
Health belief model applied to consumers and a vaccination-preventable disease

The fundamental constructs of the HBM are **perceived susceptibility** (believing one may contract a disease), **perceived severity** (believing a disease is dangerous and has serious sequelae), **perceived benefits** (believing the behaviour will effectively diminish risk or severity of a disease), **perceived barriers** (believing that obstacles to behaviour change outweigh the benefits), **cues to action** (triggering behaviour change), and **self-efficacy** (trusting one’s capacity to act). Later versions of the HBM also include the construct of *modifying factors* (socioeconomics, gender, age, personality and knowledge) which affect individuals’ beliefs and which indirectly impact their behaviour (Skinner et al. 2015). Figure 1 illustrates the proposed HBM constructs applied to consumers’ decisions on infant vaccination.

Skinner et al. (2015) criticised the HBM’s focus on cognition, which ignores the emotional component of behaviour. They also observed that, despite the frequent use of the HBM, data are still lacking on the relationships amongst the HBM constructs, with few researchers having investigated factors that could moderate the effect of the HBM’s constructs on behaviour. These limitations warrant further investigation. Moreover, from a consumer behaviour perspective, the HBM’s tendency to apply the concepts of beliefs and perceptions, without clearly defining or differentiating between these two could hinder the application in practice. Redefinition of the concepts in the HBM from a consumer behaviour perspective is thus necessary, with a view to address the factors influencing of vaccination decisions and vaccine hesitancy.

## Methodology

### Concept derivation approach

A qualitative concept derivation strategy directed by Walker and Avant (2019) was used to derive concepts of the HBM in the public health and health promotion context to be applied to the field of consumer science. The HBM was identified as the most widely used explanatory model for consumers’ vaccination decisions for this analogy, explicitly viewed from a consumer science perspective. The basis for concept derivation lies in identifying similarities to adopt novel ideas about a phenomenon. Derivation strategies can aid in developing new concepts where theory is lacking or outdated. Limitations of this process are that new concepts have limited scientific usefulness, needing verification in practice and in research (Walker & Avant 2019). Therefore, further research is needed on the connection between the derived concepts to construct an integrated theoretical model (Walker & Avant 2019).

### Steps of concept derivation

The concept derivation strategy was iterative, with four steps often co-occurring, and repetition of previous steps (Walker & Avant 2019). The **first step** required a consumer science literature review of consumers’ vaccination decisions. In **step two**, literature concerning vaccination decisions from the health sciences (public health, medicine, paediatrics, nursing, vaccinology, virology and health communication) was reviewed. **Thirdly**, concepts related to vaccination decisions from a health sciences perspective were identified. In **step four**, we redefined the HBM concepts in terms of consumer sciences.

For steps one and two, the defined topic guided the search strategy: consumers’ infant vaccination decisions. After considering all possible words, synonyms or phrases, the following search terms were included: consumer health decision/health decision influence/vaccin*/vaccine hesitant/anti-vaccin*/immune/immun*. Concepts were searched separately, and Medical Subjects Headings (MeSH) were used. Boolean operators enabled us to combine concepts and define the relationship between individual sets of concepts. Search terms were refined when searches returned very broad, untargeted results. The literature searches used PubMed, EBSCOhost, Google Scholar and Science Direct. Abstracts of search results were scanned for relevance to vaccination or primary preventive health decision influences. Additional articles by identified seminal authors not included in the initial search results were obtained.

In step three, it was found that although most models identified in consumer science literature applied to some extent, none were ideal for modelling infant vaccination decisions. Over 33 000 articles were initially collected, activating a rigorous refinement process to exclude research related to animals, veterinary sciences and laboratory research. Health science literature on vaccination decisions, although biomedical in nature, investigated health-seeking behaviour using a variety of methodologies. The HBM was selected from the health sciences to guide the selection of concepts related to vaccination decisions for the derivation process. Literature was screened for quality and bias, with selected papers being organised according to crucial HBM constructs and critically analysed through an inductive and deductive process. This strengthened the logical reasoning between theory and research, as the researcher continuously argued from evidential support (inductive) and made conclusions (deductive).

Table 1 summarises the first three steps of the concept derivation process. The results of the fourth step, redefining concepts from the HBM using a consumer sciences perspective, is presented and discussed under the results section.

**TABLE 1:** Realisation of the first three steps from the concept derivation process.

Variable	Step 1: Consumer science literature regarding consumers’ vaccination decisions	Step 2: Literature on vaccination decisions from fields other than consumer sciences	Step 3: Select concepts related to vaccination decisions from other fields
Search terms used	Consumer OR Home economics OR Family studies AND vaccin* OR immun*	Consumer AND decision, health AND decision, health decision AND influence, vaccin* OR immune* AND public health, medicine, nursing, paediatric NOT animal, veterin*	Health Belief Model, HBM AND Consumer AND vaccine* OR immune* AND Belief, value, risk, threat, barrier, benefit, consequence, information, knowledge, demograph*, personality, self-efficacy, prompt, cue NOT animal, veterin*
Search engines and databases used	PubMed, EBSCOhost, Google Scholar and Science Direct	PubMed, EBSCOhost, Google Scholar and Science Direct	PubMed, EBSCOhost, Google Scholar and Science Direct
Search refinement conducted	Scan abstracts for relevance to influences or predictors of vaccination decisions. Search in journals publishing consumer behaviour related research	Scan abstracts for relevance to influences or predictors of vaccination decisions.	Scan abstracts for relevance to influences or predictors of vaccination decisions. Identify and search for publications from seminal authors. Refer back to fundamental consumer behaviour theory

*Source*: Applied from Walker, L.O. & Avant, K.C., 2019, *Strategies for theory construction in nursing*, Pearson, London

HBM, Health belief model.

### Rigour

Trustworthiness (Guba & Lincoln 1989) was strengthened as follows: (1) To enhance credibility, the researcher reviewed the literature to become familiar with the HBM and other health behaviour-related models and consumer decision-making predictors in both consumer and health sciences. The iterative process of concept derivation combined with peer scrutiny and frequent debriefing sessions with co-authors enhanced the credibility of analysis and interpretation. (2) To enhance transferability, the researcher kept reflective notes used for clarifying the concept derivation process with co-authors. (3) The iterative process of concept derivation was recorded in detail, and a critical reflection on the appropriateness of this methodology enhanced the dependability of the study. (4) Furthermore, a detailed methodology description enhanced the confirmability of the findings.

### Ethical considerations

Approval for this study was obtained from the Scientific Committee of the Africa Unit for Transdisciplinary Health Research, and ethical approval from the Health Research Ethics Committee of the North-West University, South Africa (NWU-00104-17-A1).

## Results and discussion: Derivation of concepts from the health belief model to consumer sciences

The final step of the concept derivation process involved identifying correspondence of relevant components of the HBM with consumer behaviour components, illustrated in Table 2.

**TABLE 2:** Concepts from the health belief model transposed to influencing factors from a consumer behaviour perspective.

Concepts from the HBM	Influencing factors from a consumer behaviour perspective
Individuals’ beliefs	Consumers’ values
Perceived threat (Perceived susceptibility and perceived severity)	Risk perception (financial, physical, time, psychological and functional risk)
Perceived benefits and perceived barriers.	Consideration of future and immediate consequences.
Perceived self-efficacy	Self-efficacy
Cues to action	Cues/prompts to action
Modifying factors (age, gender, ethnicity, personality, socioeconomics, knowledge)	Demographics, personality, information and knowledge

*Source:* Applied from Rosenstock, I.M., 1974, ‘Historical origins of the health belief model’, Health Education Monographs 2(4), 328–335. https://doi.org/10.1177/109019817400200403

HBM, health belief model.

### Consumer values relating to individual beliefs from the health belief model

According to the HBM, consumers’ beliefs about their infant’s susceptibility to contracting VPDs, for example, influence their vaccination decision (Guvenc et al. 2016). Beliefs can be defined as convictions that something, in particular, is accurate or real (Sharma 2017), exposing consumers’ knowledge and evaluation of a specific object or situation (Schiffman & Wisenblit 2019). Beliefs are not necessarily based on facts or scientific evidence but stem from consumers’ experience, tradition and acquired knowledge (Ferrante-Wallace 2016). Consumers might, for example, have beliefs based on the information obtained from the internet or word-of-mouth (WOM), which is not necessarily based on scientific evidence. However, when predicting consumer behaviour, consumer scientists instead often study differences and similarities in consumer *values* (Kahle, Beatty & Homer 1986; Thienhirun & Chung 2017).

Values, which are cognitive images and principles directing thought and behaviour, incorporate attitudes and beliefs (Botha 2019; Sharma 2017). Values are more enduring and challenging to change than beliefs, as they are not tied to specific objects or situations, and they are linked with feelings that motivate action (Schwartz et al. 2012). The List of Values (LOV) developed by Maslow (1954), Rokeach (1973) and Feather (1975) identifies nine values which classify consumers based on Maslow’s hierarchy. The LOV is a useful set of predictors of behaviour (Kahle et al. 1986), corresponding with the value theory of Schwartz (1992). Because values will guide health behaviours, the influence of values on vaccination decisions warrants investigation. This is because values include the emotional component of behaviour which is lacking in the HBM (Skinner et al. 2015).

### Consumers’ risk perception relating to the health belief model’s perceived threat and perceived barriers

The HBM identifies perceived threats and perceived barriers which influence vaccination decisions. A review of 38 studies investigating the determinants of vaccination uptake divided the non-socio-demographic determinants into five groups, namely access, affordability (or constraints [Betsch et al. 2018]), awareness, acceptance and activation (Thomson, Robinson & Vallee-Tourangeau 2016). The barriers are factors contributing to risk perception, also described as uncertainty (Mishra & Das 2018) when faced with health decisions. Consumers may experience a perception of psychological (Betsch et al. 2018), functional / performance, physical, financial, social and time risk (Kaplan, Szybillo & Jacoby 1974; Schiffman & Wisenblit 2019) which may influence their vaccination decisions.

Risk perceptions may be aggravated by stories of ‘vaccine injuries’ on the internet that shape the perception of occurrence and provoke emotion (Witteman & Zikmund-Fisher 2012). Betsch et al. (2010) found that consumers who viewed typical vaccine-critical websites had an increased risk perception regarding vaccinations and decreased risk perception regarding the omission of vaccinations. The immediate risks (or perceived barriers) of vaccination are pain (physical risk), time and money spent (time and financial risk), as well as possible adverse reactions (functional, physical, psychological and possibly social risks). At the same time, the benefits are delayed and less noticeable because consumers cannot predict whether they would have contracted the disease they have been vaccinated against (Betsch & Sachse 2012). The HBM describes this uncertainty as a perceived threat (perceived severity of the VPD weighed against perceived susceptibility to contracting the VPD). Perceiving a severe threat from a VPD implies perceiving their vaccination decision to have a high immediate or future risk.

### Consideration of future consequences relating to the health belief model’s perceived threats, perceived barriers and perceived benefits

Contemplation of the HBM components of perceived threats, barriers and benefits, implicitly involves consideration of immediate consequences (e.g. pain from injection) versus future consequences (e.g. fear of future adverse reactions or protection from diseases). The construct of consideration of future consequences (CFC) refers to the differential between consideration of future against immediate consequences of behaviour (Strathman et al. 1994) often used to predict decisions (Toepoel 2010). Research shows that consumers who were more concerned with the future consequences of their actions had stronger intentions and more positive attitudes towards health-related behaviour (Joireman et al. 2012). Therefore, more significant concern about the future consequences of infant vaccination (e.g. the perceived benefit of protection against the threat of possible future disease) may outweigh concerns about perceived barriers (e.g. pain experienced from a vaccination injection), resulting in a stronger intention to vaccinate. Conversely, a perceived threat may emanate from misinformation about disabilities caused by vaccination (Larson 2018). The HBM’s perceived threats, perceived benefits and perceived barriers all relate to the CFC construct influencing health decisions. These constructs are, in turn, affected by the HBM’s modifying factors, discussed in the following section.

### Information, information sources and knowledge as influences on health decisions

Information acquisition, information sources and trust in information sources are factors influencing vaccination decisions (Dube et al. 2018). The HBM depicts knowledge (the objective and subjective assessment of the validity of information [Alba & Hutchinson 2000]) as a factor modifying health decisions. The credibility of the information source is the extent to which consumers trust and believe in the honesty, objectivity, trustworthiness and expertise of the source of the message (eds. Erasmus & Mpinganjira 2019; Schiffman & Wisenblit 2019).

Social media exposes users to internet-based vaccination-related messages and advice posted by their peers, perceived as credible information sources, as the sender seemingly has nothing to gain from the recommendation (Schiffman & Wisenblit 2019). Consequently, consumers believe like-minded peers who share messages through social media, and thereby often enhance the spread of misinformation (Johnson et al. 2020; Larson 2018). As a result, consumers might not be able to distinguish between credible and non-credible information sources (Witteman & Zikmund-Fisher 2012).

Scientific evidence contributes to consumers’ objective knowledge (Zingg & Siegrist 2012). Unfortunately, providing scientific evidence does not always convince consumers who question vaccination (Larson 2018) and, therefore, does not necessarily enhance their objective knowledge. Conversely, subjective knowledge may be based on anecdotes shared by consumers with self-reported expertise and over-confidence in what they think they know about a subject (Alba & Hutchinson 2000), resulting in a health information barrier (Noncungu & Chipps 2020). These consumers include people with medical credentials who use social media to spread misinformation or disinformation for financial gain, thereby threatening global health (Larson 2018). Unfortunately, dis- and misinformation spread more rapidly on social media than scientifically proven evidence, contributing to the domination of anti-vaccination information on these platforms (Johnson et al. 2020). Notably, both objective and subjective knowledge influence consumers’ decisions (Donoghue, Van Oordt & Strydom 2016; Dube et al. 2018). Thus, both should be considered when investigating consumers’ vaccination decisions.

### The health belief model modifying factors and demographic characteristics

The HBM also identifies personality and demographics (age, gender, ethnicity and socioeconomics) as factors modifying health decisions, including vaccination decisions (Gilkey et al. 2014). However, the demographic factors in the HBM do not include all factors which influence health decisions, such as women’s empowerment (Thorpe et al. 2016), literacy, fertility rates, and access to media (Noncungu & Chipps 2020), amongst others (Wiysonge et al. 2012). The well-established influence of personality on consumer decision-making (Matthews et al. 2017; Orji et al. 2017) allows its direct application as a consumer behaviour construct in vaccination decision influences, without derivation.

### Other health belief model concepts with direct application to consumer behaviour

Other HBM concepts are also directly applicable as influencing factors on health decisions, without redefinition. The concept of perceived self-efficacy, for example, is a well-known concept in consumer behaviour studies (Montford & Goldsmith 2016). Also, the HBM’s action includes the actual decision (whether or not to receive health interventions), with ‘cues to action’ (or prompts to action) also being familiar constructs in consumer behaviour-related studies (Delaney et al. 2017).

### Limitations

The literature search was confined to English language articles available on the specified databases. Thus, significant studies in other languages or those not indexed by these databases may have been missed. Bias in the selection of literature was limited by including experts from different fields (consumer behaviour, vaccinology and nursing) to assist in overseeing the process. Also note that the health decision as the ‘action’ part of the HBM, in this study refers to the *intention* of whether or not to vaccinate an infant. However, the actual behaviour is not implicit to the intention.

## Conclusion

To address vaccine hesitancy, an examination of consumer behaviour related to preventive primary healthcare decisions is needed. We introduced concept derivation methodology, applying and redefining the foundational HBM components to fit the field of consumer science for the specific application to consumers’ infant vaccination decisions. Consumer values are added as possible predictors of consumers’ behaviour, thereby including the affective component of behaviour lacking in the HBM. Risk perception and consideration of immediate and future consequences emerged as influencing factors, and we differentiated between consumers’ information and knowledge, which may influence their vaccination decisions.

Additionally, as an example of a primary preventive healthcare decision application, this research sets the stage for collaboration between consumer scientists and health scientists, by applying well-researched consumer behaviour concepts to gain a holistic understanding of consumers’ healthcare-related decisions. A better understanding of the influences on consumers’ primary preventive healthcare decisions can serve to promote or improve healthcare-related interventions such as infant vaccinations.

## Recommendations

The authors recommend research on consumers’ primary preventive healthcare decisions to investigate the listed factors as possible influences affecting consumers’ vaccination decisions. We acknowledge that the list of proposed factors is most likely not exhaustive, as our proposed concept derivation methodology initiates research into the underexplored field of consumer science-related primary preventive healthcare decisions. However, the factors we identified are complex and context-specific. Thus, future research should be confined to a selection of factors, guided by context-specific literature.

Our proposed concept derivation acts as a basis for further investigations. Further research needs to verify the influence of these concepts on consumers’ health decisions to describe and predict consumers’ infant vaccination decisions. Statement derivation should follow this concept derivation to test the derived concept for empirical validity and to determine the connection between the derived concepts. Finally, theory derivation should follow statement derivation to propose an altered model for predicting and explaining (Walker & Avant 2019) consumers’ infant vaccination decisions.

Finally, we highlight the opportunity for interdisciplinary research into consumer behaviour in healthcare settings, contributing to consumer well-being on a public health primary prevention level.
